# Familial Adenomatous Polyposis Manifesting as* Lactococcus* Endocarditis: A Case Report and Review of the Association of* Lactococcus* with Underlying Gastrointestinal Disease

**DOI:** 10.1155/2016/5805326

**Published:** 2016-10-12

**Authors:** Taylor C. Bazemore, Stacey A. Maskarinec, Kahli Zietlow, Edward F. Hendershot, John R. Perfect

**Affiliations:** ^1^Department of Internal Medicine, Duke University Hospital, Durham, NC, USA; ^2^Division of Infectious Diseases, Department of Internal Medicine, Duke University Hospital, Durham, NC, USA

## Abstract

A 45-year-old male with a prosthetic aortic valve presented to the hospital with several months of generalized malaise. On admission, he was noted to have anemia of unclear etiology and subsequently became febrile with multiple blood cultures growing* Lactococcus garvieae*. Inpatient workup was concerning for infectious endocarditis (IE) secondary to* Lactococcus*. The patient was discharged home with appropriate antimicrobial therapy; however, he was readmitted for persistent, symptomatic anemia and underwent colonoscopy, which revealed innumerable colonic polyps consistent with Familial Adenomatous Polyposis (FAP) that was later confirmed with genetic testing. Surveillance computed tomography (CT) imaging of the aortic repair later demonstrated valve dehiscence with surrounding fluid collection; he underwent redo surgery and was found to have destruction of the aortic annulus and a large pseudoaneurysm. Histopathology of the valve prosthesis confirmed IE. It is suspected that the patient developed* Lactococcus* IE from enteric translocation. Review of the literature provides several reports of* Lactococcus* infections in association with underlying gastrointestinal disease, including colorectal cancer. Given this association, we raise the question of whether the diagnosis of* Lactococcus* IE should evoke suspicion and encourage evaluation for gastrointestinal pathology, as occurs with* Streptococcus bovis*.

## 1. Introduction

The* Lactococcus* genus is a gram-positive, catalase-positive, anaerobic coccus that produces lactic acid from the fermentation of carbohydrates. It was formerly included in the* Streptococcus* genus and is often misidentified as* Enterococcus*. There are now eight recognized* Lactococcus* species, with the two most common being* Lactococcus garvieae* and* Lactococcus lactis*. In general, these organisms are known to be sensitive to most *β*-lactam antibiotics and aminoglycosides [1].

Although typically considered an opportunistic pathogen,* Lactococcus* has been responsible for systemic human infections with varied manifestations including bacteremia, peritonitis, liver abscess, endocarditis, and osteomyelitis [2]. Furthermore, infective endocarditis (IE) stemming from* Lactococcus* bacteremia is a particularly rare clinical entity, but it has been reported in several case studies that emphasize patients with prosthetic heart valves [3–6]. Of the reported cases of* Lactococcus* infection, there is a frequent association with consumption of raw fish or dairy products [7]. In particular,* L. garvieae* has been more broadly associated with fish and dairy consumption, with transmission also reported via contaminated water; conversely,* L. lactis* is associated primarily with dairy products [8, 9]. Importantly, in many of the reported cases, these patients had underlying gastrointestinal (GI) disease, suggesting a portal of entry [1, 5, 7, 10–13]. While not normally part of the GI microbiome,* Lactococcus* has been isolated from the intestines of humans [1, 10].

## 2. Case Presentation

A 45-year-old male was admitted for further evaluation of presumed symptomatic anemia. The patient had a past medical history significant for treatment-naïve hepatitis C, remote polysubstance abuse, and Bentall repair of an aortic root aneurysm approximately 18 months prior to presentation. He endorsed two months of generalized malaise and subjective, generalized weakness without other localizing symptoms. The patient denied consumption of raw fish or fermented milk products. Upon presentation, he was febrile to 39.5°C with other vital signs within normal limits. Physical exam was remarkable for a IV/VI systolic murmur at the left upper sternal border. Admission lab results were significant for white blood cell count of 12.9 × 10^9^/L (3.2–9.8 × 10^9^/L g/dL), hemoglobin of 10.2 g/dL (13.7–17.3 g/dL), erythrocyte sedimentation rate of 100 mm/hr (0–15 mm/hr), and C-reactive protein of 5.03 mg/dL (≤0.6 mg/dL). Blood cultures were collected, and the patient was started on empiric antibiotic therapy with vancomycin and piperacillin-tazobactam due to concern for prosthetic valve IE.

**Figure 1:**
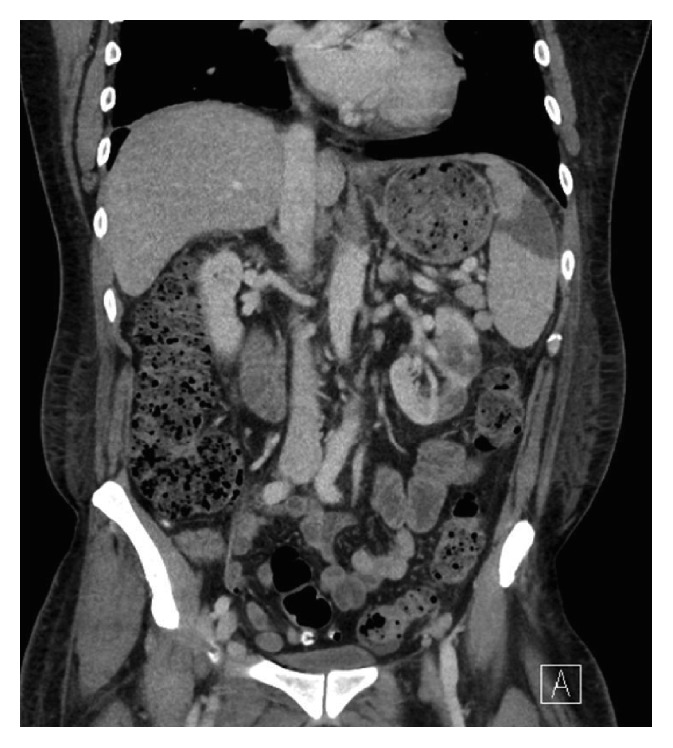
Computed tomography scan of chest/abdomen/pelvis demonstrating splenic and left renal infarction.

Admission blood cultures grew* L. garvieae* and remained positive on repeated cultures for the following three days. This pathogen was identified by matrix-assisted laser desorption/ionized time of flight (MALDI-TOF) mass spectrometry (bioMérieux Vitek MS, Knowledge Base 2.0X). Both transthoracic and transesophageal echocardiograms demonstrated thickened valvular leaflets and periaortic thickening but revealed no vegetation. CT of the chest, abdomen, and pelvis demonstrated splenic and left renal infarcts concerning for embolic phenomena (Figure 1).

On hospital day 1, the patient's hemoglobin dropped from 10.2 g/dL to 8.3 g/dL, and he continued to have persistent and worsening anemia over the course of his hospitalization. He had no signs of GI bleeding on rectal examination, and laboratory workup was consistent with anemia of chronic disease. An endoscopy was performed that was negative for any signs of bleeding but did reveal multiple duodenal polyps. A polyp was biopsied with pathology demonstrating tubular adenoma. Ultrasound of the abdomen revealed a morphologically cirrhotic liver.

The patient was diagnosed with possible prosthetic valve IE secondary to* L. garvieae* bacteremia, although he met only four of the minor Duke Criteria for endocarditis: predisposing heart valve, temperature >38°C, persistently positive blood cultures, and embolic phenomena that included infarcts of his kidney and spleen [11]. While he did not have direct or echocardiographic evidence of intracardiac infection at the time of diagnosis, the patient's clinical presentation was consistent with prosthetic valve IE, and it was recommended that he receive a six-week course of antibiotic therapy with ceftriaxone and gentamicin based on the minimal inhibitory concentration (MIC) noted on Etest. This regimen was derived from traditional recommendations for the treatment of prosthetic valve IE caused by intermediate resistance viridans group streptococci or* Streptococcus bovis* [12], although the synergistic effect of gentamycin has been proven to be limited in the treatment of* L. garvieae* [13]. The patient was only able to complete two of the six weeks of gentamicin therapy due to the development of acute kidney injury.

**Figure 2:**
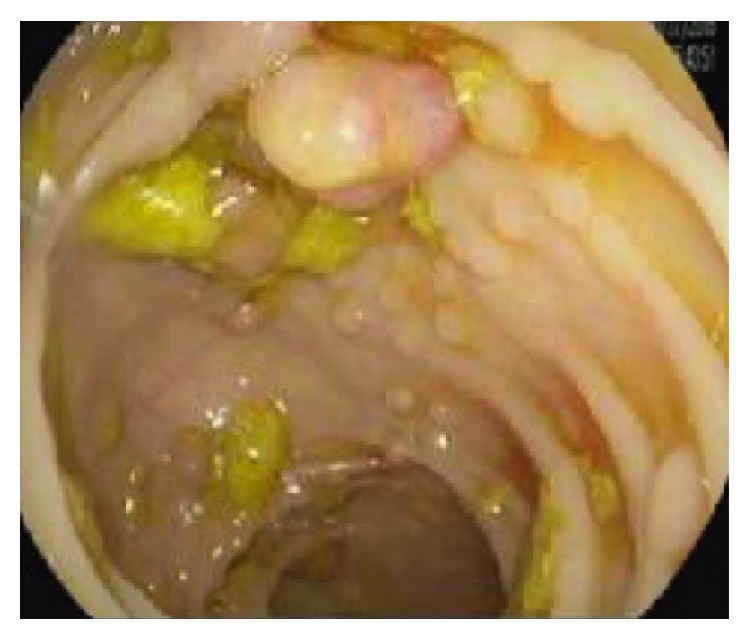
Colonoscopy demonstrating diffuse intestinal polyposis.

Nine days following his initial discharge, the patient was readmitted to the hospital after again presenting with signs and symptoms of anemia. A colonoscopy was obtained and revealed innumerable (3 to 12 mm) polyps throughout the entire colon, concerning for Familial Adenomatous Polyposis (FAP) or a similar polyposis syndrome (Figure 2). Pathology of biopsied polyps demonstrated tubular adenomas and tubulovillous adenomas with high-grade dysplasia but no evidence of invasive carcinoma. Given the extent of his polyposis, the patient was advised to undergo definitive surgical management; he ultimately underwent total colectomy 8 months following his initial presentation. Subsequent genetic testing revealed that the patient was positive for the mutated* APC* gene thereby confirming the diagnosis of FAP [23].

Following completion of six weeks of antibiotic therapy, the patient underwent surveillance CT imaging that demonstrated partial aortic valve dehiscence and a fluid collection surrounding the aortic valve prosthesis concerning for pseudoaneurysm. Given these findings, the patient was referred for surgical management. Four months following his initial presentation, he underwent repair of the aortic root with replacement of the bioprosthetic valve. Intraoperatively, there was near-complete dehiscence of the valve conduit from the annulus and significant destruction of the aortic annulus with a large pseudoaneurysm. Cultures of the valve were negative for bacterial growth and pathology showed chronic inflammatory changes without any signs of residual infection. These operative findings are nonetheless indicative of a prior intracardiac infection, satisfying the remainder of the Duke Criteria and thus confirming his diagnosis of prosthetic valve IE secondary to* L. garvieae* bacteremia.

The patient tolerated the surgery and remained stable through the postoperative period. He was discharged home on postoperative day 5. Following discharge, the patient was free from any signs or symptoms of persistent or relapsed infection. He underwent surveillance cardiac magnetic resonance imaging (MRI) at 14 months following surgery which showed no abnormalities of the repaired aortic root or aortic valve prosthesis.

## 3. Discussion

The patient in this reported case presented with anemia of unclear etiology and was ultimately found to have diffuse intestinal polyposis concerning for FAP. Genetic testing revealed that the patient was heterozygous for a pathogenic variant in the* APC* gene, consistent with FAP or attenuated FAP. Revelation of the patient's extensive GI pathology occurred in conjunction with his diagnosis of* L. garvieae* endocarditis. This patient lacked the traditional risk factors of raw fish or dairy consumption that have been reported to predispose patients to infection with* Lactococcus*. It is suspected that bacterial translocation associated with his colonic disease likely facilitated this infection. Notably, given the patient's history of polysubstance abuse, it is important to consider intravenous (IV) drug use as an alternative mode of bacterial introduction; however, the patient denied any current or prior use of IV drugs. Furthermore,* Lactococcus* IE has not been described to occur in association with IV drug use.

Given the development of a pseudoaneurysm in the setting of the patient's bacteremia, it is important to consider mycotic aneurysm as the primary nidus of infection, as* Lactococcus* IE has been reported to occur in the association with mycotic aneurysms [5]. However, the operative findings and pathological analysis of the pseudoaneurysmal tissue did not show any signs of infection consistent with mycotic aneurysm, although surgery was performed following the completion of antibiotic therapy. The patient's concomitant hepatitis C cirrhosis may have acted as an additional risk factor for the development of IE, considering the increased risk of bacteremia in patients with cirrhosis secondary to the compromise in host defense [24, 25]. Zuily and colleagues similarly describe a patient with* Lactococcus* IE in the setting of hepatitis C cirrhosis [22].

Several prior clinical reports describe* Lactococcus* infections in association with underlying GI diseases, including cases of patients with colonic polyps or colorectal cancer, as well as patients with nonneoplastic lesions such as diverticular and ulcerative disease; we have reviewed the literature for cases of* Lactococcus* infections in association with GI diseases (Table 1). Reports include patients who are mostly of middle-to-advanced age with a variety of GI pathologies, but to date, this is the first case of* Lactococcus* infection that has been reported in association with FAP or other diffuse GI polyposis syndromes.

In this case, the diagnosis of* L. garvieae* prosthetic valve IE proved to be a harbinger of a serious underlying disease. Considering his significant polyp burden and concomitant symptomatic anemia, it is possible that a timely colonoscopy would have otherwise revealed this polyposis syndrome. However, in patients with less severe disease, the diagnosis of* Lactococcus* bacteremia and/or endocarditis may be an early indication of undiagnosed GI pathology, including colonic malignancy. Therefore, given our findings and other reports of* Lactococcus* infections associated with GI lesions including colorectal cancer, we propose that the diagnosis of* Lactococcus* endocarditis should evoke suspicion and encourage evaluation for GI pathology as is recommended with* Streptococcus bovis* IE [26]. By establishing the association between this infection and the potential risk of underlying GI lesions including colorectal carcinoma, expedient colonoscopy in patients with* Lactococcus* endocarditis may allow for earlier diagnosis and treatment of cancer in patients with occult disease.

## Figures and Tables

**Table 1:** Review of reported *Lactococcus* infections in association with underlying gastrointestinal disease.

Reference	Age/sex	Type of infection	Preexisting GI lesion(s)	Predisposing risk factors	Treatment	Outcome
Antolín et al. [14]	79/F	Liver abscess	Diverticulosis	None reported	Imipenem-cilastatin (5 w)	Clinical improvement
Chan et al. [15]	70/M	Infectious spondylodiscitis	Gastritis	Raw fish consumption	Ampicillin (6 w)	Clinical improvement
Fihman et al. [16]	86/F	Prosthetic AV IE	Duodenal ulcer	Prosthetic AV and cholecystectomy	Amoxicillin, gentamicin, (4 w) and then amoxicillin (3 w)	Clinical improvement
Fleming et al. [3]	68/M	Prosthetic AV IE and native MV IE	Colon polyps	Raw fish consumption and prosthetic AV	Vancomycin (6 w)	Clinical improvement
Kim et al. [17]	69/M	Acalculous cholecystitis	Gastric ulcer	Raw fish consumption, working as fisherman, and alcoholism	Cefminox and then cefaclor (8 d)	Clinical improvement
Mofredj et al. [18]	68/F	Liver abscess	Cholangiocarcinoma	Biliary prosthesis and steroid use	Amoxicillin, netilmicin, and metronidazole (12 d until death)	Died from GI hemorrhage
Nadrah et al. [19]	81/M	Bacteremia	Diverticulosis	Prosthetic AV, prosthetic MV, and PPM	Piperacillin-tazobactam, then ampicillin, and gentamicin (6 w)	Clinical improvement
Ortiz et al. [20]	77/F	Native MV/AV IE	Colorectal carcinoma	Recent colorectal carcinoma surgery	Ampicillin and gentamicin (6 w)	Died from heart failure
Rasmussen et al. [5]	81/M	Prosthetic AV IE and native MV IE	Diverticulosis	Prosthetic AV	Penicillin and tobramycin (3 w)	Clinical improvement
Vinh et al. [1]	80/M	Native AV IE	Colon polyp	None reported	Ampicillin (6 w)	Clinical improvement
Wang et al. [21]	72/M	MV IE	Gastric ulcer	Raw fish consumption	Penicillin (4 w) and gentamicin (2 w)	Clinical improvement
Wang et al. [21]	56/F	Bacteremia	Small bowel diverticulosis	None reported	Cefazolin, gentamicin (2 d), and then cotrimoxazole (5 d)	Clinical improvement
Wang et al. [21]	47/M	Peritonitis	Intestinal perforation	Raw fish consumption	Piperacillin and amikacin (1 w)	Clinical improvement
Zuily et al. [22]	64/F	Prosthetic MV IE	Colon polyps	Fish consumption, prosthetic MV, PPM, and cirrhosis	Amoxicillin and gentamicin (6 w)	Clinical improvement

AV = aortic valve, MV = mitral valve, TV = tricuspid valve, IE = infective endocarditis, PPM = permanent pacemaker, w = week, d = day, F = female, and M = male.
